# Particle Engineering of Chitosan and Kaolin Composite as a Novel Tablet Excipient by Nanoparticles Formation and Co-Processing

**DOI:** 10.3390/pharmaceutics13111844

**Published:** 2021-11-03

**Authors:** Chonwipa Yarangsee, Phanphen Wattanaarsakit, Jakkapan Sirithunyalug, Phuriwat Leesawat

**Affiliations:** 1 Department of Pharmaceutical Sciences, Faculty of Pharmacy, Chiang Mai University, Chiang Mai 50200, Thailand; chonwipa_y@payap.ac.th (C.Y.); jakkapan.s@cmu.ac.th (J.S.); 2 Department of Pharmaceutics and Industrial Pharmacy, Faculty of Pharmaceutical Sciences, Chulalongkorn University, Bangkok 10330, Thailand; aphanphe@chula.ac.th

**Keywords:** co-processing, chitosan, kaolin, nanoparticles, spray drying

## Abstract

Chitosan is not a common excipient for direct compression due to poor flowability and inadequate compressibility. Co-processing of chitosan and kaolin is a challenging method to overcome the limitations of the individual excipients. The purpose of the present study was to develop co-processed chitosan–kaolin by the spray drying technique (rotary atomizer spray dryer) and to characterize the excipient properties. The formation of chitosan nanoparticles was the major factor for desirable tablet hardness. The ratio of chitosan/tripolyphosphate of 10:1 and 20:1 had a significant effect on hardness. The successful development of co-processed chitosan–kaolin as a novel tablet excipient was obtained from a feed formulation composed of chitosan and kaolin at a ratio of 55:45 and the optimum chitosan/tripolyphosphate ratio of 20:1. Co-processing altered the physical properties of co-processed chitosan–kaolin in such a way that it enhanced the flowability and tableting performance compared to the physical mixture.

## 1. Introduction

Tablets have been an attractive choice for pharmaceutical manufacturing over several decades and are a commonly used dosage form for drug administration. Consideration of the tableting approach is dependent on the characteristic properties of the active pharmaceutical ingredient (API), the excipients, and their stability during the manufacturing process [1]. Direct compression (DC) is the preferable method for tablet preparation since it requires fewer processing steps, avoids exposure to heat and moisture, resulting in good stability of the therapeutic drug [2], and offers lower production costs. Nevertheless, API that can be processed into tablets by DC are limited due to poor flowability and compactibility properties. Thus, excipients that enhance powder flowability and compactibility are required in order to increase the use of DC in tablet production and ensure robust manufacturing [1]. Although, many DC carriers are commercially available, most of them cannot be classified as multifunctional excipients; thus, several excipients have to be used in a DC formulation in the tablet manufacturing industry.

Currently, particle engineering in the pharmaceutical industry has become an important topic to control a range of key unit manufacturing operations [3]. It requires an understanding of particle formation processes. Particle engineering combines many elements of science including chemistry, formulation science, colloid and interface science, heat and mass transfer, solid state physics, aerosol and powder science, and nanotechnology [4].

The co-processing method is applied to improve the functionality of an existing excipient by combining parent excipients with other tablet excipients using an appropriate process [5,6]. Many excipients can be engineered as co-processed excipients to provide the desired properties to be used in DC [7]. The co-processed excipients are physically modified without chemical structure alteration while they synergistically increase their functional performances [8,9]. Thus, a combination of existing excipients, using processing techniques, is an interesting option for new excipient development, which can be brought to market without undergoing the safety and toxicity testing of a completely new chemical.

Plastic and brittle materials are usually employed in the co-processing method to enhance excipient functionality [9,10]. Existing excipients of reasonable price and ready availability such as chitosan (plastic material) and kaolin are remarkable choices for particle engineering using the co-processing technique. Chitosan has been accepted as an effective tablet disintegrant due to its high water absorption capacity [11]. The excellent swelling capacity of chitosan in water was also indicated in a previous study by Rasool et al. [12]. Kaolin has been used in many pharmaceutical applications as an excipient or active ingredient, because it exhibits excellent surface physicochemical properties and is stable material. Nevertheless, chitosan powder shows poor flow ability of pharmaceutical blends in large-scale production [13,14], while tablets containing kaolin as a diluent present poor mechanical strength. A report by Badwan et al. [13] also pointed out the poor compressibility of chitosan, resulting in tablets with low crushing strength due to the high porosity of chitosan powder.

Co-processed excipients can be produced by many different processing methods. This study concentrates on spray drying, which is the process of choice for well-known commercial co-processed excipients. Spray drying is a widely used technique for novel pharmaceutical excipient development, as it can provide spherical particles in a narrow particle size range including the free flowing agglomerates that are suitable for direct compression method [15,16]. Furthermore, spray drying offers several advantages including good reproducibility and, if rotary atomizer is used, air dispersion creates a high degree of rotation, resulting in a uniform drying temperature [4].

Chitosan nanoparticles preparation was reported in several studies which apply a fundamental of chitosan nanoparticles to the innovative platform for natural molecules or drug delivery [17,18,19] including a cooperation between nano/microparticles formation with spray drying [20]. Chitosan cross-linking with sodium tripolyphosphate (STPP) are widely used for biomedical and pharmaceutical applications since STPP is classified by the Food and Drug Administration as being a Generally Recognized as Safe Substance (GRAS) [20].

In the present study, co-processing of chitosan–kaolin via spray drying was aimed at providing superior properties to these compounds compared to the individual excipients or their physical mixture. Chitosan solution converted to chitosan nanoparticles prior to combination with kaolin was found to overcome the drawbacks of the existing chitosan powder in tablet production. The effect of STPP on morphology, flow properties, and tablet hardness of the co-processed excipients were investigated. The functionality of the resulting co-processed chitosan–kaolin as a novel tablet excipient was evaluated, in comparison with commercial co-processed excipients (Avicel SMCC90^®^).

## 2. Materials and Methods

### 2.1. Materials

Chitosan (squid chitosan, product code SQA190) was purchased from Marine Bio Resources Co., Ltd. (Bangkok, Thailand). Kaolin was obtained from Creative Industrial Material Research and Development Center, Lampang city, Thailand. Silicified microcrystalline cellulose; Avicel SMCC90^®^ (product code S71704C, FMC International Company, Little Island, Cork, Ireland) was purchased from Onimax Co., Ltd., Bangkok, Thailand. Sodium tripolyphosphate (CAS No. 7758-29-4, product code 12321JI-057) was a product of Sigma–Aldrich, Darmstadt, Germany. Glacial acetic acid (CAS No. 64-19-7, product code 155220) was a product of QRec chemical, Auckland, New Zealand. All other chemicals used were of analytical reagent grade or equivalent.

### 2.2. Preparation of Co-Processed Chitosan-Kaolin (CCK) Using the Spray Drying Technique

#### 2.2.1. CCK Preparation

Spray drying of the feed suspension was performed using a Niro rotary atomizer spray dryer (serial no.2648, GEA Niro, Copenhagen, Denmark). The inlet temperature, atomizing air pressure, and feed flow rate were set at 160 °C, 1.2 bar, and 10 mL/min, respectively (from the preliminary study of spray drying parameters). Kaolin was added to chitosan nano/microparticles with constant stirring, and the feed suspension was stirred until the end of spray drying process. The properties of CCK were evaluated including particle morphology, flowability and compression behavior.

#### 2.2.2. Selection of an Optimum Feed Formulation

Chitosan solution of 1% *w*/*v* concentration was prepared by adding a quantity of chitosan 10 g to 1 L of 2% (*v/v*) acetic acid with constant stirring until a complete solution was obtained. Sodium tripolyphosphate was dissolved in water and TPP solution of 1% *w*/*v* concentration was added dropwise into the chitosan solution under magnetic stirring at room temperature to obtain chitosan nano/microparticles, and then further stirred for 30 min. Kaolin was gradually blended with chitosan nano/microparticles prior to spray drying. The ratios of chitosan/TPP were selected from the preliminary study. Feed formulations for the spray drying process are shown in Table 1. The effect of the chitosan/kaolin ratio and chitosan/TPP ratio, based on dry chitosan weight, were evaluated with regard to flow properties and particle bonding strength (tablet hardness).

The size of chitosan micro/nanoparticles was observed by particle size analyzer (Zetasizer ZS, Malvern Instruments Ltd., Malvern, UK). Optimum feed formulation providing satisfaction of pharmaceutical excipient properties of CCK was further characterized and compared with Avicel SMCC90^®^.

### 2.3. Co-Processed Chitosan-Kaolin (CCK) Characterization

#### 2.3.1. Flow Properties

Flow properties were evaluated via the determination of the angle of repose (AR) and compressibility index (CI) on the freshly spray-dried samples (with a moisture content between 4% and 5%) to minimize the effect of moisture content on flowability. AR was manually measured using the fixed funnel method [21]. Bulk density is the ratio of weight to the volume of sample. Carefully level the sample powder with constant weight (10 g) into a dry 25 mL graduated cylinder and read the apparent volume to the nearest graduated unit. The cylinder was then mechanically tapped 1250 times using a Jolting volumeter (Stav 2003, Erweka, Langen, Germany) to obtain the tapped volume. Tapped density is determined as the weight of the sample to the volume after tapping a measuring cylinder. An average of three determinations (*n* = 3) of all tests was recommended. CI was calculated from the bulk and tapped densities using the equation;

% CI = [(Tapped density– Bulk density)/Tapped density] × 100.

#### 2.3.2. Loss on Drying

The residual moisture of sample in terms of percentage loss on drying (% LOD) was carried out by a moisture balance, Sartorius MA-50 moisture analyzer (Sartorius company, Goettingen, Germany). The co-processed chitosan–kaolin (approximately 1 g) was accurately weighed on to a sample pan and placed in the moisture analyzer. An infrared energy heater was used to heat the sample. The temperature was brought up to 105 °C. As the sample is heated, it loses moisture. The loss of moisture translates to a loss of weight of the sample. When the weight of the sample no longer changes, the instrument shuts off the heat. Moisture is calculated automatically by comparing the initial sample weight to the dried or final sample weight. The test was repeated in triplicate (*n* = 3).

#### 2.3.3. Tablet Hardness

The sample powder was compressed with an 11.0 mm diameter of a flat-faced punch under the compression pressure of 98 MPa for 10 s, using a hydraulic press machine (C, Carver, Wabash, IN, USA). The average tablet hardness was evaluated for 10 tablets (*n* = 10).

#### 2.3.4. Scanning Electron Microscopy (SEM)

The shape and surface morphology of co-processed chitosan–kaolin was determined by scanning electron microscopy (JSM-IT300, Joel Ltd., Tokyo, Japan). The sample was sputtered with gold prior to SEM examination.

#### 2.3.5. Powder X-ray Diffraction

The XRD patterns of samples were recorded using powder X-ray diffractometer (D8, Bruker, Bremen, Germany) with a reflection mode (at 40 kV, 40 mA over the range of 5°–80° 2 ϑ using Cu Ka radiation wavelength of 1.5406 Å) to determine the crystalline structure of co-processed chitosan–kaolin, those of the individual excipients, and spray dried chitosan nanoparticles.

#### 2.3.6. Powder Characteristics

Particle size analysis of co-processed chitosan–kaolin was performed using laser diffraction particle size analyzer (Mastersizer S, Malvern Instruments Limited, Worcestershire, UK). Sample was dispersed in ethanol for the measurement. The values of D10, D50, D90, and mean diameter of the particle size distribution were recorded.

Single station automatic gas pycnometer (AccuPyc II 1340, Micromeritics, Norcross, GA, USA) was operated for true density measurement of powders (co-processed chitosan-kaolin, Avicel SMCC90^®^ and physical mixture of chitosan and kaolin). A sample was dried at 60 °C for 24 h before the investigation. True density was calculated using the gas displacement method. Powder was placed in the sample cup, followed by purging with helium gas at 25–30 °C 10 times.

### 2.4. Evaluation of Co-Processed Excipient Properties

#### 2.4.1. Compression Behavior

##### Tablet Preparation

A hydraulic press machine and a flat-faced punch (11.0 mm diameter) were used to produce the tablets. The powder was compressed for 10 s at 98, 147, and 196 MPa compression pressure. Each tablet was accurately weighed, with a diameter (mm) and thickness (mm) was determined using a Vernier caliper. The data were recorded to analyze the compression behavior.

##### Tablet Tensile Strength

A tablet breaking force (PTB-311 Pharmatest, Hainburg, Germany) was used to determine the breaking force of the compacts. The tablet tensile strength can then be calculated using Fell and Newton’s method [22];

σ_x_ = 2 X/dt

where σ_x_ is the tensile strength (MPa), X is the hardness (N), d is the diameter of the compact (mm), and t is the thickness of the compact (mm).

#### 2.4.2. Disintegration Property

Tablets of excipients were prepared using a hydraulic press machine at 98 MPa compression pressure. Disintegration test was performed of six tablets (*n* = 6) according to the standard USP method [23] with a disintegration apparatus (Erweka ZT122, Erweka GmbH, Langen, Germany). Disintegration time was recorded when all of the tablets had disintegrated completely.

### 2.5. Statistical Analysis

All tests were carried out at least in triplicate and expressed as mean ± standard deviation (SD). SPSS (version 17.0, IBM, Chicago, IL, USA) was employed to carry out one-way analysis of variance (ANOVA). Statistical significance between the factor in respond to physical property of co-processed excipient was performed using Tukey’s honestly significant difference (HSD) multiple range test at a 95% confidence level.

## 3. Results and Discussion

### 3.1. Preparation of Co-Processed Chitosan-Kaolin (CCK) Using the Spray Drying Technique

The size of chitosan micro- or nanoparticles formed depended on the chitosan to TPP ratio (Figure S1, Tables S1 and S2). Chitosan solution crosslinked with TPP was observed by particle size analyzer (Zetasizer ZS, Malvern Instruments Ltd., Malvern, UK) (Figures S2 and S3). The results show that chitosan nanoparticles were obtained when the formulation consisted of a chitosan to TPP ratio of 20:1. The average size of chitosan nanoparticles was 223.2 ± 12.7 nm (PI 0.81) and the average zeta potential was +78.8 mV. On the other hand, chitosan microparticles were detected at the lower chitosan to TPP ratio of 10:1, the average size was 3545.5 ± 1765.8 nm (PI 0.76, the average zeta potential was +76.3 mV). This may have contributed to the smaller particle sizes found in the presence of a lower amount of TPP [24,25].

In general, chitosan solution becomes opalescent when chitosan nanoparticles are formed and progressively more turbid when the amount of TPP increases. A higher amount of TPP generally leads to a higher mean particle size of chitosan nanoparticles. The particle size also depends on the amount of chitosan in the feed. Therefore, chitosan nanoparticles that are in the nano-size range occur at the optimal ratio of TPP and chitosan due to the fact that the chitosan molecules are fully crosslinked. On the other hand, as the amount of TPP continues to increase above the optimum chitosan/TPP ratio, chitosan nanoparticle size starts to increase due to particle agglomeration [25,26]. Hence, an excess of TPP at the particle surface can crosslink the particles between one another, resulting in nanoparticles becoming microparticles. The physical properties of co-processed chitosan-kaolin with the effect of TPP might be explained by chitosan nanoparticles or microparticles formation.

As seen in Table 2, the non-crosslinked formulations (control), the feed containing 100% of chitosan and kaolin. Spray dried kaolin showed poor flowability. Moreover, spray dried powder of chitosan and kaolin exhibited very low tablet hardness (approximately 5–6 kg). Hence, the physical properties of CCK were directly improved by chitosan-TPP crosslinking and spray drying.

#### 3.1.1. Effect of the Chitosan/Kaolin Ratio and the Chitosan/TPP Ratio on Flow Properties

Various ratios of TPP were evaluated with different amounts of chitosan. The results show that the flow character of the CCK of R1–R5 was poor and the compressibility index value was greater than 27%, as shown in Table 2. The results also indicate that increasing the TPP mass ratio together with feed formulations containing more than 50% chitosan resulted in the worst flow properties of CCK, classified as very to extremely poor. As mentioned above, the effect of TPP on the flow character of CCK demonstrated that TPP with lower amounts of chitosan (R1, R2) led to better flowability.

**Figure 1:**
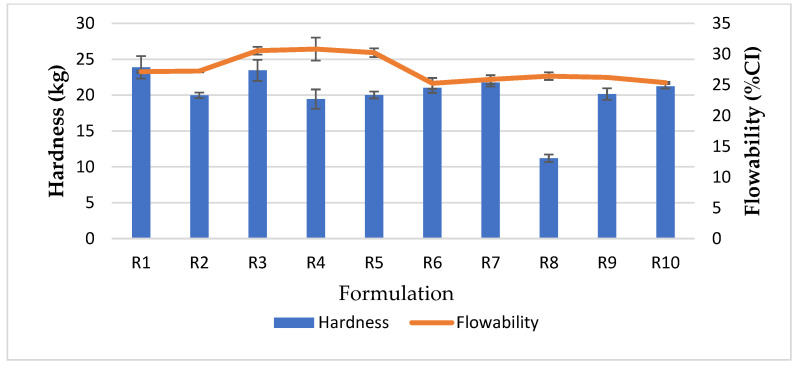
Tablet hardness versus flow properties of CCK.

Hydrated kaolin was added to the feed formulation of R6 instead of dry kaolin; the flow properties of CCK were significantly improved with an angle of repose indicating excellent flowability compared to the CCK of R5. The flowability improvement of CCK R7–R10 was consistent with R6 (Figure 1), in which the feed contained hydrated kaolin.

#### 3.1.2. Effect of the Chitosan/Kaolin Ratio and the Chitosan/TPP Ratio on Tablet Hardness

Co-processed chitosan–kaolin obtained from chitosan–TPP nanoparticles could provide a very high tablet hardness under pressure of 98 MPa, as shown in Table 2. Chitosan/TPP mass ratios of 10:1 and 20:1 improved the tablet hardness, the results indicate that the optimal ratio between chitosan and TPP provided a fully nano/microparticles formation. Thus, the large surface area of the sub-micron sized spray dried powder was available for particle–particle bonding to form a closely packed structure under tablet compression.

Feed formulations containing hydrated kaolin also provide the maximum tablet hardness of CCK dramatically and improved the flow properties. This can be explained by the hydration capacity of kaolin [27]. The results of the infrared study show that the inner OH groups of kaolinite/kaolin are disturbed because of the partial collapse of the hydrated structure and channeling of water molecules and/or other small molecules into the kaolin structure [27,28]. Thus, chitosan nanoparticles (particle size average 223.2 nm) might insert into porous structure of hydrated kaolin (particle size average 14.8 µm), resulting in the creation of a network between chitosan and kaolin.

In addition, the chitosan/TPP ratio used in the crosslink interaction can affect the tablet hardness. The results indicate that the optimum ratio of chitosan/TPP was 20:1, whereas a lower ratio of chitosan/TPP (10:1) led to lower tablet strength, as did the chitosan/kaolin ratio of 55:45. Besides the tablet hardness of co-processed chitosan–kaolin, tablet disintegration was likewise considered; the disintegration time significantly decreased (*p* < 0.05, one-way ANOVA) with an increase in the amount of chitosan to 55% (R7, R8) as shown in Table 3. Rapid tablet disintegration of co-processed of R7 and R8 was explained by the capillary action of chitosan [29] and swelling capacity of chitosan and kaolin. Kaolin has little capacity to swell in water [30]. Furthermore, chitosan nanoparticles homogeneously distributed in the porous structure of kaolin, could continue to take up water, then thoroughly spread it via percolation through tubes that work by capillary action, leading to swelling and rapid tablet disintegration.

**Figure 2:**
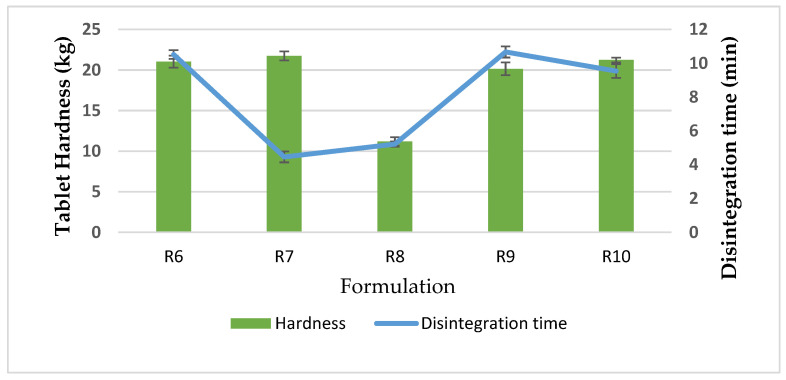
Tablet hardness and disintegration time of CCK R6–R10.

The chitosan to kaolin ratio was adjusted by varying the amount of chitosan at 50:50, 55:45, 45:55 and 40:60, respectively (R6, R7, R9, R10); the chitosan/TPP ratio was fixed at 20:1. The physical properties of flow character and tablet hardness were similar. Of note, the compressed tablets of R7 disintegrated quickly (within 5 min) although the tablet strength was not significantly different, as shown in Figure 2.

As mentioned above, the results show that the optimal ratio of chitosan to kaolin (as hydrated kaolin) was 55:45 and a chitosan/TPP ratio at 20:1 can enhance the physical properties of the resulting CCK prepared by spray drying, in terms of flowability and tablet hardness.

### 3.2. Co-Processed Chitosan–Kaolin (CCK) Characterization

**Figure 3:**
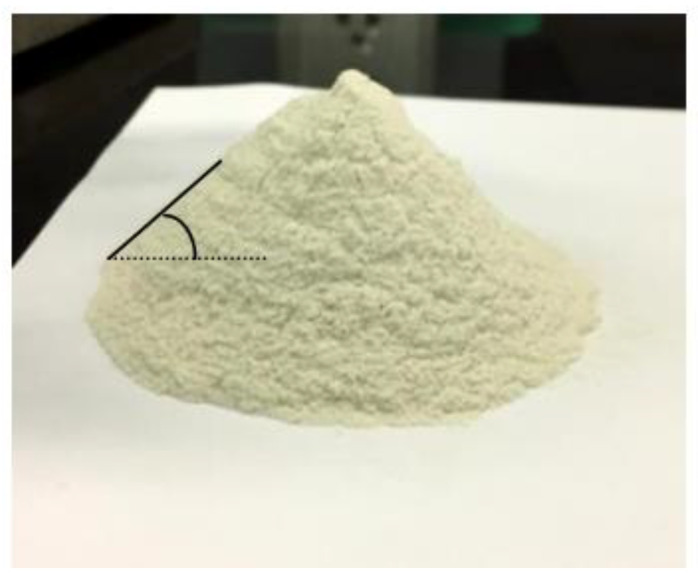
Appearance of co-processed chitosan–kaolin R7.

Co-processed chitosan–kaolin R7 (CCK-R7) was considered the most suitable formulation for a novel tablet excipient as it showed significantly enhance tablet hardness, true density, powder flowability and disintegration time, compared to the other formulations. Co-processed chitosan–kaolin R7 contained 55% chitosan (chitosan/TPP mass ratio 20:1) and 45% kaolin. The powder was slightly yellowish. In the fixed funnel method, CCK-R7 was poured through a funnel to form cone, the angle of the resulting cone was directly measured as illustrated in Figure 3. Spray drying was continuously operated to produce the CCK-R7 for further evaluation and compared with Avicel SMCC90^®^.

#### 3.2.1. Morphology and Particle Size

**Figure 4:**
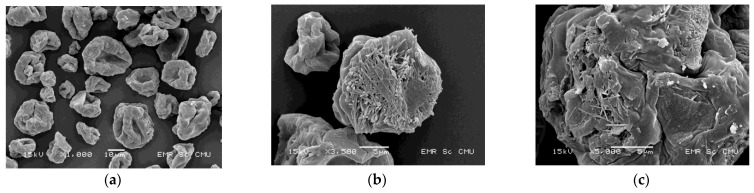
Scanning electron photomicrographs of CCK-R7 at magnification of (a) 1000×, (b) 3500× and (c) 5000×.

The shape and surface topography of CCK-R7 were examined by SEM at different magnifications. The morphology of the spray dried powder is presented in Figure 4. The particles showed an irregular shape, rough surface and chitosan filaments were observed on the outer layer of the particles. Chitosan particles crosslinked with TPP was a critical factor leading to particle deformation. Previous studies [25,31] also reported that the TPP cross-linking interaction affects the particle morphology of spray dried chitosan–TPP, in which the higher cross-linking ratio resulted in a smoother surface with a buckled structure or collapsed characteristic of skin-forming spray dried materials.

Particle formation by the spray drying method occurred upon hot air-droplet contact, as fast water or solvent diffusion from the droplet core to its surface allowed for constant moisture removal. The formation mechanism is described in terms of the Peclet number, which is influenced by a combination properties of the solute and solvent. The ratio between the diffusional motion of the solutes and the droplet evaporation rate was regarded, and low density particle morphology was explained by concept of the Peclet number [4]. In case of a high Peclet number (>1), the droplet surface becomes elevated with the ingredient and suspended material may form a composite shell. The diffusion rate of the solute is lower than droplet evaporation rate. Hence, the resulting particles can have different morphologies, dependent on their sizes and the properties of their shells in the final stage of the drying process. If the shells become rigid quickly, a hollow sphere can be formed. Otherwise, particle shrinkage or dimpled particles are formed [4,32].

**Figure 5:**
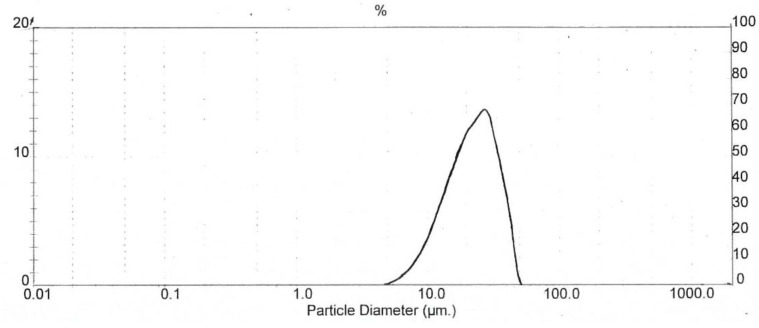
Particle size distribution of CCK-R7.

The particle size distribution curve exhibits the various size particles and the distribution of particle size range. Figure 5 represents the distribution of particles in relation to particle diameter versus the percentage of total particle volume of co-processed R7. The highest dispersion (~70%) is produced by particles of 15–30 µm. The particles had a volume mean diameter (D_4,3_), median particle size (d_50_), and span value of 23.38, 22.46 and 1.119, respectively. The spray drying technique for CCK-R7 preparation provided particles with a narrow size distribution, which can be observed by the narrow distribution curve. Particle sizes of CCK-R7 were in the range of 5.45–51.62 µm. The size and shape of particles had a direct influence on the physical properties of the materials [33]. Since irregular shaped and fine particles of CCK-R7 were found, the effect of particle size and shape of CCK-R7 on flow properties will be further investigated.

#### 3.2.2. Powder X-ray Diffractometry

**Figure 6:**
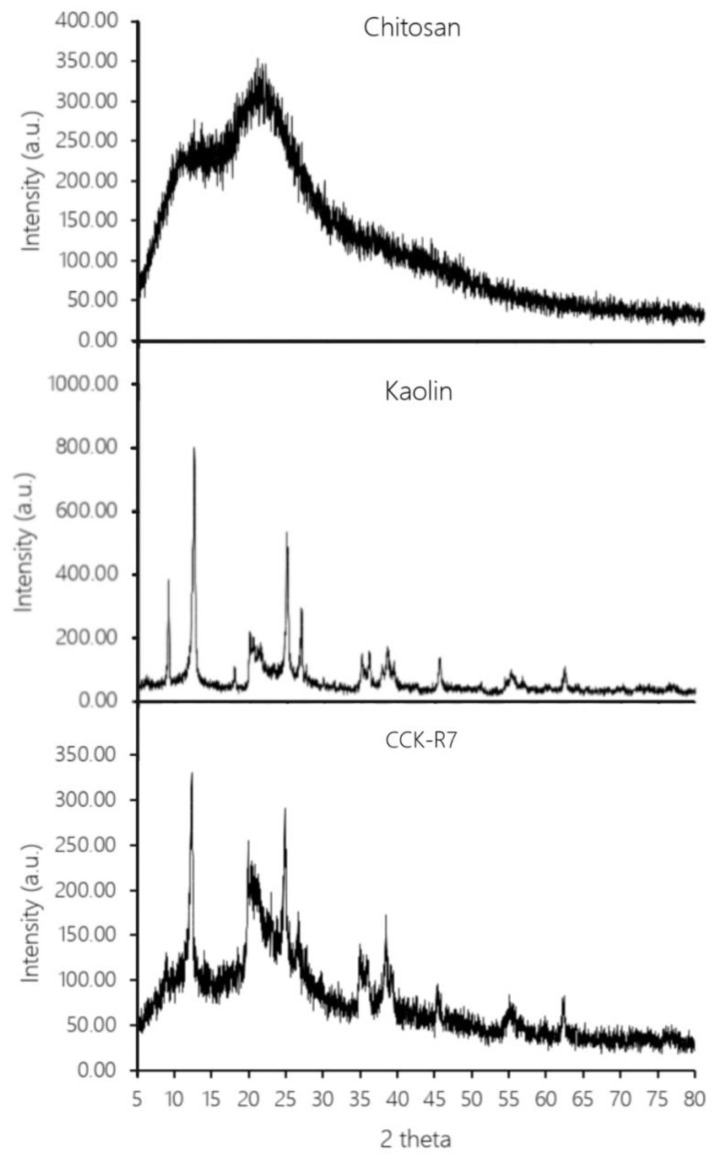
X-ray diffraction patterns of chitosan, kaolin and CCK-R7.

Powder X-ray powder diffractometry (PXRD) was used for solid state identification of chitosan, kaolin and CCK-R7. The transformation of excipients using spray drying was also evaluated by PXRD. The PXRD pattern of chitosan powder showed peaks associated with an amorphous solid while the pattern of a crystalline state was observed in kaolin. Figure 6 reveals that no unusual peaks were evident when comparing the PXRD patterns of CCK-R7 with chitosan and kaolin powder. It can be concluded that the interaction between chitosan and TPP and hydrated kaolin followed by the spray drying process did not affect the solid state modification of the developed CCK-R7.

In addition, the crystallinity together with amorphous of CCK-R7 affected the compaction behavior of the material, as the hardness of CCK-R7 tablets was greater than that of individual chitosan and kaolin. From X-ray diffraction pattern of sample, the crystallite size estimation can calculate by Scherer equation that relates the size of sub-micrometer crystallite in a solid.

### 3.3. Powder Characterization and Compaction of Co-Processed Excipients

#### 3.3.1. Powder Flow Properties

Powder flow properties were measured in terms of the angle of repose, compressibility index, Hausner ratio, flow rate and true density. The flow properties of CCK-R7, Avicel SMCC90^®^, and the physical mixture of chitosan and kaolin were investigated, as shown in Table 4 and Table 5.

Co-processed excipients such as co-processed R7 and Avicel SMCC90^®^ revealed greater flowability compared to the physical mixture of chitosan and kaolin. The flow properties of Avicel SMCC90^®^ were improved by the coating of silicon dioxide on the particle surface using the spray drying technique [4]. Although, the particles of Avicel SMCC90^®^ are not spherical in shape, the larger particle sizes result in superior powder flowability. On the other hand, the irregular shaped and fine particles of CCK-R7, with particle sizes in the range of 5.45–51.62 µm, led to a flow character of CCK-R7 that was not comparable with Avicel SMCC90^®^.

The true density of powders often differs from that of the bulk material because the process manufacturing changes the physical structure and therefore the density of each particle in a powder. Whereas bulk and tapped density are dependent on the particle size as the lower particle size results in higher bulk and tapped density, the flowability characteristics of a powder are directly related to powder density. Furthermore, bulk density is an indicator of powder flowability to undergo compression and compaction in which a powder with very low bulk density may require densification before tableting [16]. The true and bulk densities of Avicel SMCC90^®^ were lower than those of CCK-R7 and the physical mixture of chitosan and kaolin. As an explanation, a coarse, homogenous spray dried powder has a lower bulk density than a fine homogenous powder such as the physical mixture of chitosan and kaolin. These results correspond to several studies showing that powders with low bulk density produced by spray drying show improvements in flowability [16,34].

#### 3.3.2. Pressure-Hardness Profile Determination

The compactibility of samples was evaluated based on the hardness of the tablets. The optimum compression force can provide excellent tablet hardness. Hardness assesses the endurance of tablets to withstand breakage, chipping, or friability under the conditions of storage, transportation, and handling [1]. Generally, tablet hardness is dependent on the type and concentration of the binder, tablet diameter, and compression force.

**Figure 7:**
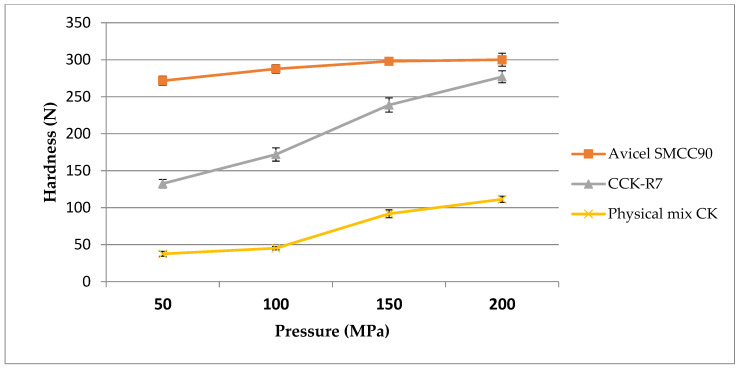
The hardness pressure profile of Avicel SMCC90^®^, CCK-R7, and the physical mixture of chitosan and kaolin.

In the DC method, the composition and type of the excipient are major factors that influence tablet performances, including the tablet hardness. Figure 7 shows the effect of the excipients on the hardness when compressed at different compression pressures. The hardness pressure profile of Avicel SMCC90^®^ demonstrated that it could give the maximum tablet strength even at a low compression force, followed by a region with a minimal increase in tablet hardness as the compression force was increased.

The compaction profile of CCK-R7 was more linear and tablet hardness continued to increase as the compression force was increased, up to the maximum compression force examined. Meanwhile, the tablet strength of the physical mixture of chitosan and kaolin was extremely low at a low compression force. Although tablet hardness increased at high compression forces; however, the tablet strength was significantly lower than those formed with Avicel SMCC90^®^ and CCK-R7. This was mainly attributed to the plastic deformation and high bond-formation ability of the co-processed excipients [35].

#### 3.3.3. Tablet Tensile Strength

**Figure 8:**
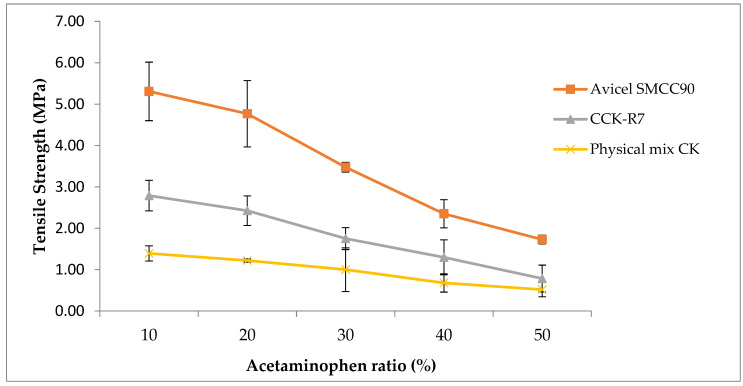
Tablet tensile strength versus the percentage of acetaminophen using Avicel SMCC90^®^, CCK-R7, and the physical mixture of chitosan and kaolin.

Acetaminophen was used as a model drug and tablet strength was considered as a major indicator for future tablet preparation. The tensile strength versus the percentage of acetaminophen were plotted. The results of the dilution potential study indicate that, as the percentage of acetaminophen increased, the crushing strength decreased (Figure 8).

The physical mixture of chitosan and kaolin showed the lowest tablet strength while Avicel SMCC90^®^, a commercially available co-processed excipient, provided the maximum tablet tensile strength at all dilution levels. The optimal concentration of acetaminophen for CCK-R7 was in range of 10–30% at which the tensile strength was more than 2 MPa; this provided better tablet hardness (approximately 10 kg) when compared to the physical mixture of the individual excipients. Thus, the spray drying process altered the physicomechanical properties of CCK-R7 in such a way that it enhanced the flowability of materials and compaction behavior.

## 4. Conclusions

The successful development of co-processed chitosan–kaolin as a novel tablet excipient was obtained from a feed formulation composed of 55% chitosan and 45% kaolin at the optimum chitosan/TPP ratio of 20:1. Kaolin was prepared as a hydrated material which was allowed to swell in water before being added to chitosan nanoparticles. The homogeneous distribution of chitosan nanoparticles into the hydrated kaolin structure in the feed suspension led to enhance flowability of co-processed chitosan–kaolin and provided the optimum tablet hardness, along with rapid disintegration. Although the CCK-R7 characteristics were not be comparable with those of Avicel SMCC90^®^, the physical properties and tablet performances were improved compared to the physical mixture of the individual materials.

Based on the results of this study, it may be concluded that it is possible to improve the compressibility and flowability of chitosan and kaolin by the co-processing method while maintaining the good properties of the original chitosan material required for a multifunctional tablet excipient that could be applied in direct compression.

Although the developed CCK-R7 presented the good appearances and physical characteristics, critical parameters should be further carefully evaluated in future development including the adjustment of spray drying for scale-up production, and the tableting behavior in simulated industrial compression conditions. In addition, particle engineering is promising direction and should be further encouraged for pharmaceutical excipient development.

## Figures and Tables

**Table 1:** Feed formulations used in the spray drying process.

Feed Formulations	Chitosan/Kaolin Ratio	Chitosan/TPP Ratio
Chitosan	100:0	-
Kaolin	0:100	-
R1	30:70	10:1
R2	30:70	20:1
R3	70:30	10:1
R4	70:30	20:1
R5	50:50	20:1
R6	50:50	20:1
R7	55:45	20:1
R8	55:45	10:1
R9	45:55	20:1
R10	40:60	20:1

R1–R5: dry powder kaolin, R6–R10: kaolin was swelled in water (hydrated kaolin).

**Table 2:** Physical properties of the developed co-processed chitosan–kaolin.

Feed Formulations	Physical Properties of CCK			
	Compressibility Index (%)	Angle of Repose (°)	Loss on Drying (%)	Tablet Hardness (kg)
Chitosan	25.02 ± 0.89	33.25 ± 1.95	5.34 ± 0.25	6.87 ± 0.29
Kaolin	30.81 ± 2.01	38.01 ± 0.25	5.81 ± 0.66	5.64 ± 0.66
R1	27.14 ± 0.19	32.55 ± 1.18	4.91 ± 0.44	23.87 ± 1.57
R2	27.24 ± 0.18	36.42 ± 1.26	5.02 ± 0.32	19.97 ± 0.39
R3	30.56 ± 0.62	45.50 ± 1.24	5.44 ± 0.30	23.45 ± 1.48
R4	30.82 ± 1.87	46.75 ± 1.20	5.35 ± 0.47	19.44 ± 1.35
R5	30.23 ± 0.71	41.75 ± 1.17	5.12 ± 0.35	20.01 ± 0.47
R6	25.25 ± 1.11	30.76 ± 1.22	4.95 ± 0.34	21.03 ± 0.55
R7	25.88 ± 0.46	31.58 ± 1.18	4.98 ± 0.49	21.74 ± 0.52
R8	26.42 ± 0.43	32.54 ± 1.10	5.03 ± 0.30	11.20 ± 0.87
R9	26.22 ± 0.44	33.15 ± 1.44	5.16 ± 0.29	20.15 ± 0.73
R10	25.35 ± 0.35	31.67 ± 1.87	5.06 ± 0.39	21.23 ± 0.30

R1–R5: dry powder kaolin, R6–R10: hydrated kaolin.

**Table 3:** True density and tablet disintegration of CCK of R6–R10.

Feed Formulations	True Density (g/cm^3^)	Disintegration Time (min)
R6	1.7222 ± 0.0007	10.52 ± 0.26
R7	1.7142 ± 0.0004	4.46 ± 0.32
R8	1.6947 ± 0.0009	5.22 ± 0.15
R9	1.7942 ± 0.0010	10.67 ± 0.33
R10	1.7967 ± 0.0010	9.54 ± 0.41

**Table 4:** Corresponding flowability parameters (USP 42) of three types of excipients.

Excipients	Angle of Repose (deg)	Compressibility Index (%)	Hausner Ratio
Avicel SMCC90^®^	Excellent (25.33)	Fair (17.92)	Fair (1.22)
CCK-R7	Good (31.58)	Passable (25.45)	Passable (1.34)
Physical mixture	Fair (35.17)	Poor (30.19)	Very poor (1.48)

**Table 5:** Flow properties of three types of excipients.

Parameters	Avicel SMCC90^®^	CCK-R7	Physical Mixture of Chitosan and Kaolin
Angle of repose (°)	25.33 ± 0.88	31.58 ± 1.18	35.17 ± 0.52
Compressibility index (%)	17.92 ± 0.54	25.45 ± 0.01	30.19 ± 0.01
Hausner ratio	1.22 ± 0.01	1.34 ± 0.02	1.48 ± 0.02
Flow rate (g/s)	0.502 ± 0.001	0.433 ± 0.002	0.347 ± 0.005
True density (g/cm^3^)	1.5982 ± 0.0008	1.7141 ± 0.0007	1.8278 ± 0.0008
Bulk density (g/mL)	0.37 ± 0.003	0.47 ± 0.024	0.51 ± 0.008

## Data Availability

Data is contained within the article.

## Supplementary Materials

The following are available online at www.mdpi.com/article/s1, Figure S1: Scanning electron photomicrographs of chitosan micro/nanoparticles crosslinked with sodium tripolyphosphate, Table S1: Size of chitosan nanoparticles crosslinked with TPP at ratio of chitosan/TPP 20:1, Figure S2: Particle size distribution curve of chitosan nanoparticles crosslinked with TPP at ratio of chitosan/TPP 20:1, Table S2: Size of chitosan microparticles crosslinked with TPP at ratio of chitosan/TPP 10:1, Figure S3: Particle size distribution curve of chitosan microparticles crosslinked with TPP at ratio of chitosan/TPP 10:1.

## Abbreviations

API	Active pharmaceutical ingredient
CCK	Co-processed chitosan-kaolin
CI	Compressibility index
DC	Direct compression
STPP	Sodium tripolyphosphate
