# Supplementary material for: Particle Engineering of Chitosan and Kaolin Composite as a Novel Tablet Excipient by Nanoparticles Formation and Co-Processing
Source: Pharmaceutics. 2021 Nov 3;13(11):1844. doi: 10.3390/pharmaceutics13111844 (PMC8618914; doi:10.3390/pharmaceutics13111844)
Supplement: Supplementary file 1 [file pharmaceutics-13-01844-s001.zip › pharmaceutics-1379946-supplementary.pdf]

pharmaceutics

MDPI

Supplementary Materials: Particle Engineering of Chitosan and Kaolin Composite as a Novel Tablet Excipient by Nanoparticles Formation and Co-processing

Chonwipa Yarangsee, Phanphen Wattanaarsakit, Jakkapan Sirithunyalug and Phuriwat Leesawat

Chitosan micro/nanoparticles characteristic

The samples were studied using SEM to determine morphology. Because of the instrument limitation, in which sample must be prepared as a dried sample prior examination. Sample was centrifuged at 3,000 rpm for 5 min. The particles that settle out from the system become sediment. The sediment was dried under 80 °C until the dried sample obtained. The dried sample was ground into a very fine powder prior SEM examination.

Many filaments of chitosan were observed in Figure S1 (a) at ratio of chitosan/TPP 10:1, this reason may rely on the particle aggregation or fusion that became the enlarged and reshaped particle. Particle size of the samples was not significant different as seen in Figure S1 (b) - (d) thus, the actual size of chitosan micro- or nanoparticles was proved by particle size analyzer (Zetasizer).

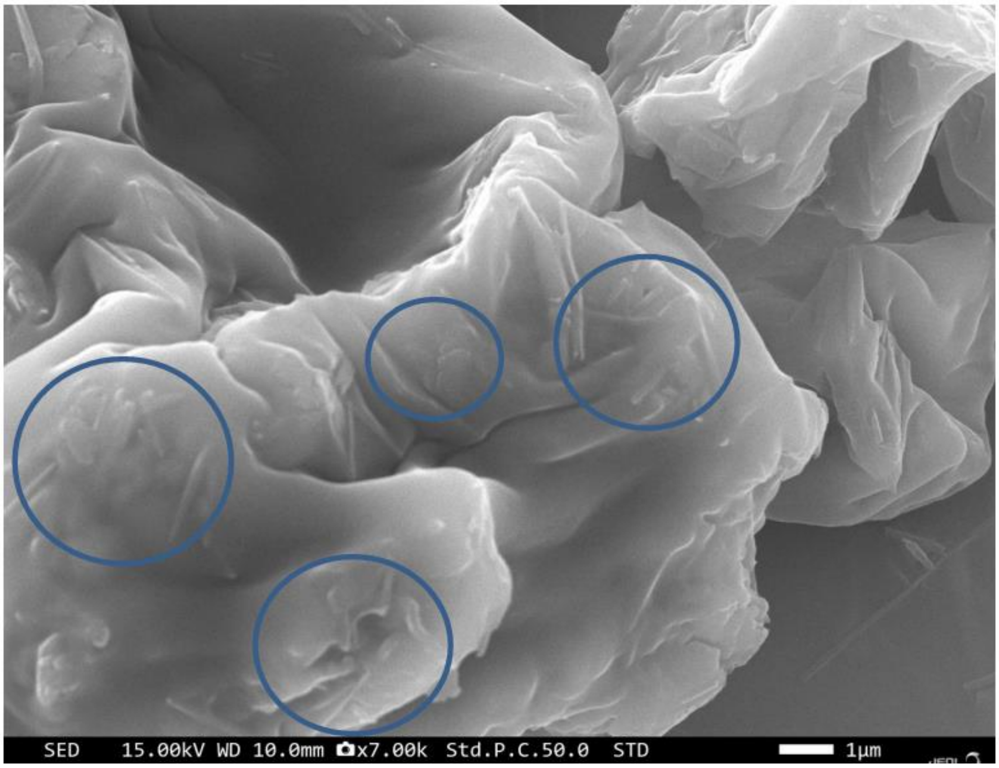

(a)

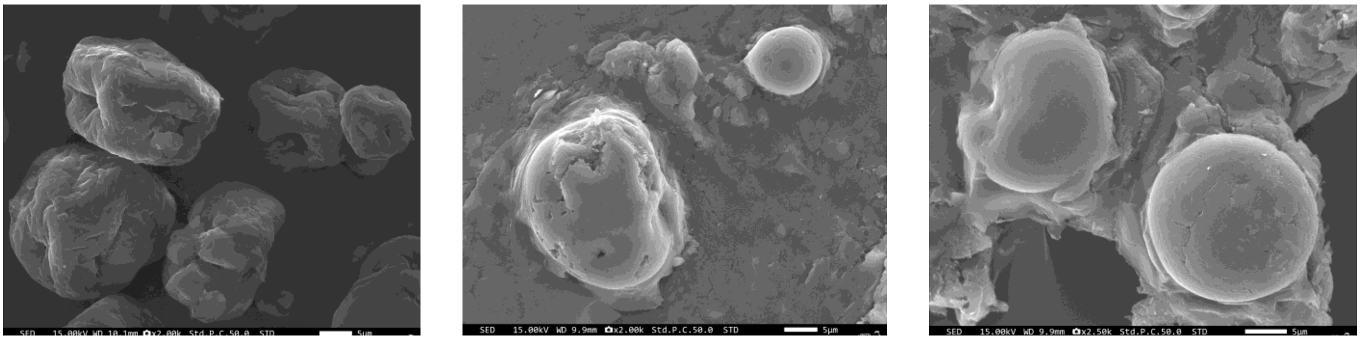

(b) (c) (d)

Figure S1. Scanning electron photomicrographs of chitosan micro/nanoparticles crosslinked with sodium tripolyphosphate; (a), (b) ratio of chitosan/TPP 10:1 and (c), (d) ratio of chitosan/TPP 20:1.

Particle size analysis

The size of chitosan micro- or nanoparticles was observed by particle size analyzer (Zetasizer ZS, Malvern Instruments Ltd., UK) as shown in Table S1 and S2, and particle size distribution curves of chitosan nanoparticles and microparticles were displayed in Figure S2 and S3, respectively.

Table S1. Size of chitosan nanoparticles crosslinked with TPP at ratio of chitosan/TPP 20:1.

No.	Diameter	Frequency	Cumulation	No.	Diameter	Frequency	Cumulation
1	0.34	0.000	0.000	43	57.09	0.000	0.000
2	0.38	0.000	0.000	44	64.50	0.000	0.000
3	0.43	0.000	0.000	45	72.87	0.000	0.000
4	0.49	0.000	0.000	46	82.33	0.000	0.000
5	0.55	0.000	0.000	47	93.02	0.000	0.000
6	0.62	0.000	0.000	48	105.10	0.000	0.000
7	0.70	0.000	0.000	49	118.74	0.000	0.000
8	0.80	0.000	0.000	50	134.16	0.000	0.000
9	0.90	0.000	0.000	51	151.57	0.000	0.000
10	1.02	0.000	0.000	52	171.25	0.000	0.000
11	1.15	0.000	0.000	53	193.48	0.000	0.000
12	1.30	0.000	0.000	54	218.60	34.377	34.377
13	1.47	0.000	0.000	55	246.96	65.623	100.000
14	1.66	0.000	0.000	56	279.04	0.000	100.000
15	1.87	0.000	0.000	57	315.27	0.000	100.000
16	2.11	0.000	0.000	58	356.20	0.000	100.000
17	2.39	0.000	0.000	59	402.44	0.000	100.000
18	2.70	0.000	0.000	60	454.69	0.000	100.000
19	3.05	0.000	0.000	61	513.71	0.000	100.000
20	3.45	0.000	0.000	62	580.41	0.000	100.000
21	3.89	0.000	0.000	63	655.76	0.000	100.000
22	4.40	0.000	0.000	64	740.89	0.000	100.000
23	4.97	0.000	0.000	65	837.07	0.000	100.000
24	5.61	0.000	0.000	66	945.74	0.000	100.000
25	6.34	0.000	0.000	67	1068.52	0.000	100.000
26	7.17	0.000	0.000	68	1207.24	0.000	100.000
27	8.10	0.000	0.000	69	1363.97	0.000	100.000

28	9.15	0.000	0.000	70	1541.04	0.000	100.000
29	10.34	0.000	0.000	71	1741.10	0.000	100.000
30	11.88	0.000	0.000	72	1967.14	0.000	100.000
31	13.20	0.000	0.000	73	2222.51	0.000	100.000
32	14.91	0.000	0.000	74	2511.05	0.000	100.000
33	16.84	0.000	0.000	75	2837.04	0.000	100.000
34	19.03	0.000	0.000	76	3205.35	0.000	100.000
35	21.50	0.000	0.000	77	3621.48	0.000	100.000
36	24.29	0.000	0.000	78	4091.63	0.000	100.000
37	27.45	0.000	0.000	79	4622.81	0.000	100.000
38	31.01	0.000	0.000	80	5222.96	0.000	100.000
39	35.03	0.000	0.000	81	5901.02	0.000	100.000
40	39.58	0.000	0.000	82	6667.10	0.000	100.000
41	44.72	0.000	0.000	83	7532.65	0.000	100.000
42	50.53	0.000	0.000	84	8510.56	0.000	100.000

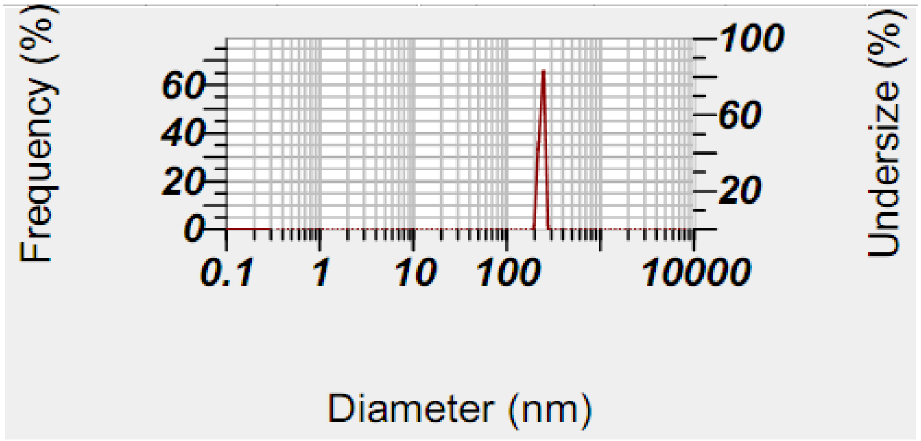

Figure S2. Particle size distribution curve of chitosan nanoparticles crosslinked with TPP at ratio of chitosan/TPP 20:1.

Table S2. Size of chitosan microparticles crosslinked with TPP at ratio of chitosan/TPP 10:1.

No.	Diameter	Frequency	Cumulation	No.	Diameter	Frequency	Cumulation
1	0.34	0.000	0.000	43	57.09	0.000	0.000
2	0.38	0.000	0.000	44	64.50	0.000	0.000
3	0.43	0.000	0.000	45	72.87	0.000	0.000
4	0.49	0.000	0.000	46	82.33	0.000	0.000
5	0.55	0.000	0.000	47	93.02	0.000	0.000
6	0.62	0.000	0.000	48	105.10	0.000	0.000
7	0.70	0.000	0.000	49	118.74	0.000	0.000
8	0.80	0.000	0.000	50	134.16	0.000	0.000
9	0.90	0.000	0.000	51	151.57	0.000	0.000
10	1.02	0.000	0.000	52	171.25	0.000	0.000
11	1.15	0.000	0.000	53	193.48	0.000	0.000
12	1.30	0.000	0.000	54	218.60	34.377	34.377
13	1.47	0.000	0.000	55	246.96	65.623	100.000
14	1.66	0.000	0.000	56	279.04	0.000	100.000
15	1.87	0.000	0.000	57	315.27	0.000	100.000

16	2.11	0.000	0.000	58	356.20	0.000	100.000
17	2.39	0.000	0.000	59	402.44	0.000	100.000
18	2.70	0.000	0.000	60	454.69	0.000	100.000
19	3.05	0.000	0.000	61	513.71	0.000	100.000
20	3.45	0.000	0.000	62	580.41	0.000	100.000
21	3.89	0.000	0.000	63	655.76	0.000	100.000
22	4.40	0.000	0.000	64	740.89	0.000	100.000
23	4.97	0.000	0.000	65	837.07	0.000	100.000
24	5.61	0.000	0.000	66	945.74	0.000	100.000
25	6.34	0.000	0.000	67	1068.52	0.000	100.000
26	7.17	0.000	0.000	68	1207.24	1.008	1.008
27	8.10	0.000	0.000	69	1363.97	2.863	3.871
28	9.15	0.000	0.000	70	1541.04	4.758	8.629
29	10.34	0.000	0.000	71	1741.10	6.277	14.906
30	11.88	0.000	0.000	72	1967.14	7.277	22.183
31	13.20	0.000	0.000	73	2222.51	7.769	29.952
32	14.91	0.000	0.000	74	2511.05	7.842	37.793
33	16.84	0.000	0.000	75	2837.04	7.623	45.416
34	19.03	0.000	0.000	76	3205.35	7.249	52.665
35	21.50	0.000	0.000	77	3621.48	6.846	59.511
36	24.29	0.000	0.000	78	4091.63	6.519	66.031
37	27.45	0.000	0.000	79	4622.81	6.344	72.374
38	31.01	0.000	0.000	80	5222.96	6.363	78.737
39	35.03	0.000	0.000	81	5901.02	6.594	85.331
40	39.58	0.000	0.000	82	6667.10	7.028	92.359
41	44.72	0.000	0.000	83	7532.65	7.641	100.000
42	50.53	0.000	0.000	84	8510.56	0.000	100.000

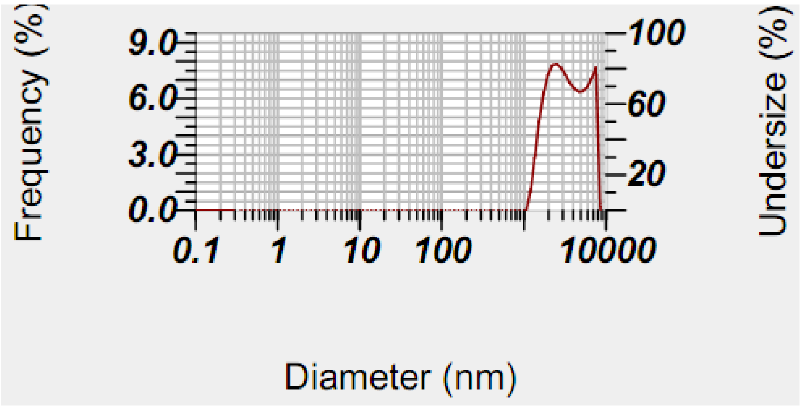

Figure S3. Particle size distribution curve of chitosan microparticles crosslinked with TPP at ratio of chitosan/TPP 10:1.
